# Relationship of liver fat content with systemic metabolism and chronic complications in patients with type 2 diabetes mellitus

**DOI:** 10.1186/s12944-023-01775-6

**Published:** 2023-01-24

**Authors:** Weiwei Ren, Yunlu Feng, Youzhen Feng, Jiaying Li, Chuangbiao Zhang, Lie Feng, Lijuan Cui, Jianmin Ran

**Affiliations:** 1 Department of Endocrinology and Metabolism, The First Affiliated Hospital of Jinan University, No.613, West Huangpu Avenue, Huizhou District, Guangzhou, 510630 China; 2 General Practice Department, South China Normal University Hospital. No.55, West of Zhongshan Avenue, Tianhe District, Guangzhou, 510632 China; 3 Medical Imaging Center, The First Affiliated Hospital of Jinan University, No.613 West Huangpu Avenue, Tianhe District, Guangzhou, 510000 China; 4 Department of Endocrinology and Metabolism, Guangzhou Baiyun District Maternity and Child Healthcare Hospital, No.1128 Airport Road, Guangzhou, 510000 China; 5 Department of Endocrinology and Metabolism, Guangzhou Red Cross Hospital Affiliated to Jinan University, No. 396, Tongfu Middle Road, Huizhou District, Guangzhou, 510220 China

**Keywords:** Type 2 diabetes mellitus, IDEAL-IQ, Liver fat content, Metabolic disorder, Chronic complications

## Abstract

**Objective:**

This study investigated the correlation of liver fat content (LFC) with metabolic characteristics and its association with chronic complications in type 2 diabetes mellitus (T2DM) patients.

**Methods:**

Eighty-one prospectively enrolled T2DM patients were divided into non-alcoholic fatty liver disease (NAFLD) group and the non-NAFLD group according to the presence of NAFL complications. LFC was determined by MRI IDEAL-IQ Sequence, and patients were divided into 4 groups according to LFC by quartile method. Basic information, metabolic indexes, and occurrence of chronic complications in different groups were analyzed and compared.

**Results:**

BMI, SBP, DBP, TG, ALT, AST, GGT, UA, HbA1c, FCP, 2 h CP, HOMA-IR, and HOMA-IS in the NAFLD group were significantly higher than the non-NAFLD group (*P* < 0.05). The incidences of chronic complications in the NAFLD group were higher than in the non-NAFLD group but not statistically significant (*P* > 0.05). BMI, SBP, DBP, TC, TG, ALT, AST, FCP, 2 h CP, HOMA-IR, and HOMA-IS showed significant differences between the patients with different LFC, and these indexes were significantly higher in patients with higher LFC than those with lower LFC (*P* < 0.05). Moreover, diabetes duration, TC, HOMA-IR, and LFC were the risk factors for ASCVD complications, while diabetes duration, TG, and LDL-C were risk factors for DN complications. Also, diabetes duration and SBP were risk factors for both DR and DPN complications in T2DM patients (*P* < 0.05).

**Conclusion:**

LFC is positively correlated with the severity of the systemic metabolic disorder and chronic complications in T2DM patients.

## Introduction

Type 2 diabetes mellitus (T2DM) is a metabolic disease characterized by increased blood glucose levels resulting from disturbances of insulin secretion, insulin action, or both [1]. It is estimated that T2DM will account for about 90% of 642 million individuals suffering from diabetes worldwide by 2040 [2]. With the increased incidence of obesity, many studies have demonstrated the contribution of fatty acids to the development of T2DM [3]. It has been reported that the prevalence of T2DM in obese adults is three to seven times higher than in normal-weight adults [4]. Normally, fatty acids produced by metabolism in the body are predominantly stored in adipose tissue in the form of triglycerides [5]. However, T2DM patients, in addition to hyperglycemia, always invariably manifest a serious breakdown in lipid dynamics, which often is reflected by higher levels of circulating free fatty acids [6]. When the rate of fatty acid production overloads the adipose tissue adaptation, the fatty acid can be heterotopically deposited in non-fat tissues and organs, such as the liver, muscle, and pancreas [3]. Fat deposition in the liver may induce insulin resistance and a rise in blood sugar levels, which in turn may lead to T2DM [5]. On the other hand, insulin resistance, in turn, can facilitate excess liver fat deposition, oxidative hepatocellular damage, inflammation, and activation of fibrogenesis that eventually develops into cirrhosis and hepatocellular carcinoma [7]. Non-alcoholic fatty liver disease (NAFLD) has been reported to have a close relationship with insulin resistance and T2DM [8]. Studies have shown that intra-abdominal fat accumulation (mainly liver fat deposition) plays a key role in the occurrence and development of insulin resistance, which promotes T2DM development and other associated chronic complications through direct or indirect mechanisms [9–11]. However, the diagnosis of NAFLD was typically based on ultrasonographic findings in most previous studies, which could not precisely quantify LFC. Therefore, the accurate determination of liver fat content (LFC) is of great importance.

With the continuous progress of technology, magnetic resonance examination plays a major role in the microscopical quantitative evaluation of fatty liver at the cellular and molecular level, which has many advantages including non-ionizing radiation, non-invasion, high resolution of soft tissue, and multiple positions imaging. 1H-MRS has been considered the "gold standard" for non-invasive quantitative diagnosis of fatty liver disease [12] and is widely used to determine the severity of fatty liver in a number of large clinical studies. However, 1H-MRS is time-consuming and requires professional knowledge analysis, which is generally only provided in academic centers, thus limiting its wide application in clinical practice. In 2005, Reeder et al. first proposed the iterative decomposition of water and fat with echo asymmetry and least squares estimation (IDEAL) [13, 14], which has been developed into a new technology of iterative decomposition of water and fat with echo asymmetry and least square estimation-iron quantification (IDEAL-IQ). IDEAL-IQ technology can collect six echo signals in one echo using three-dimensional fast spoiled gradient recalled echo (FSPGR) and estimate complex field mapping with the iterative least square method. MRI IDEAL-IQ Sequence is characterized by its simple operation, three-dimensional scan of the whole liver, and accurate water–lipid separation images, which can directly measure the LFC percentage value of the region of interest (ROI) without complicated calculations. MRI IDEAL-IQ Sequence is not only equivalent to 1H-MRS result in the quantitative diagnosis of LFC but also positively correlated with liver biopsy [15]. In addition, MRI IDEAL-IQ Sequence is also a volumetric scan and can be used for quantitative measurement of the whole liver based on voxel fat quantification at the same time. It has been reported that even in the presence of metal ions, the effect of water–lipid separation with IDEAL-IQ is accurate and reliable, thus liver steatosis can be accurately and quantitatively diagnosed [16].

As mentioned above, 1H-MRS is the "gold standard" for quantitative diagnosis of fatty liver disease, and MRI IDEAL-IQ Sequence, as the latest, convenient technology best correlated with MRS results, has resolved the problem of accurate quantification of visceral fat content. Here, our objective was to introduce the MRI IDEAL-IQ Sequence technology for the first time to precisely determine the LFC in T2DM patients and then explored the correlation of LFC with metabolic characteristics and T2DM-associated chronic complications.

## Materials and methods

### Participants

A total of 81 patients, diagnosed with T2DM according to the World Health Organization diagnostic criteria, were prospectively enrolled from August 2014 to May 2015 at our hospital. The exclusion criteria were as follows: (1) patients with type 1 diabetes mellitus, diabetes complicated with pregnancy, or other diabetes types; (2) patients having acute complications of diabetes; (3) patients with a history of liver disease; (4) huge liver cysts and hemangiomas, which affect the delineation of ROI; (5) patients receiving drugs that contribute to liver fat deposition; (6) patients with thyroid disease; and (7) patients with severe systemic diseases. This study was approved by the Ethics Committee and Review Board of our hospital. All patients enrolled in this study provided informed consent, and the study was conducted ethically in accordance with the World Medical Association Declaration of Helsinki.

Patients were grouped in two ways. Group 1: The LFC cutoff value of 5.56% determined by MRI IDEAL-IQ Sequence was used as the basis for grouping, thus patients were divided into the NAFLD group with LFC ≥ 5.56% and non-NAFLD group with LFC < 5.56% [17]. Group 2: After measuring LFC using MRI IDEAL-IQ Sequence, patients were divided into four groups (group A, group B, group C, and group D) from high to low LFC using P25, P50, and P75 as the critical value.

### Clinical data and examination indexes

The clinical data of patients were recorded by questionnaire. The parameters included were; age, sex, height, weight, body mass index (BMI), diabetes duration, systolic blood pressure (SBP), diastolic blood pressure (DBP), diabetes medication history, other medical histories, family disease history, etc. After fasting and water deprivation for more than 8 h, blood was collected intravenously for biochemical examination, including glutamic-pyruvic transaminase (ALT), glutamic oxalacetic transaminase (AST), gamma-glutamyl transpeptidase (GGT), alkaline phosphatase (ALT), serum creatinine (Scr) for calculating estimated glomerular filtration rate (eGFR), blood urea nitrogen (BUN), Cystatin C (CYC), uric acid (UA), apolipoprotein A (ApoA), apolipoprotein B (ApoB), lipoprotein (a), triglyceride (TG), total cholesterol (TC), glycosylated hemoglobin (HbA1c), high-density lipoprotein cholesterol (HDL-C), low-density lipoprotein cholesterol (LDL-C), urine creatinine and urinary microalbumin for calculating albumin-to-creatinine ratio (ACR). Islet cell function-related indexes, including fasting blood glucose (FPG), fasting C-peptide (FCP), 2 h postprandial blood glucose (2 h PG), 2 h postprandial C-peptide (2 h CP) were detected to calculate homeostasis model assessment of insulin resistance (HOMA-IR) and homeostasis model assessment of insulin secretion (HOMA-IS). Enzyme-linked immunosorbent assay kits were used to determine the inflammation markers. Imaging examinations, including coronary artery CT, coronary angiography, cephalic magnetic resonance arteriography, carotid color ultrasonography, arteriovenous ultrasonography of both lower extremities, and abdominal ultrasound were performed.

### MRI IDEAL-IQ sequence

**Fig. 1:**
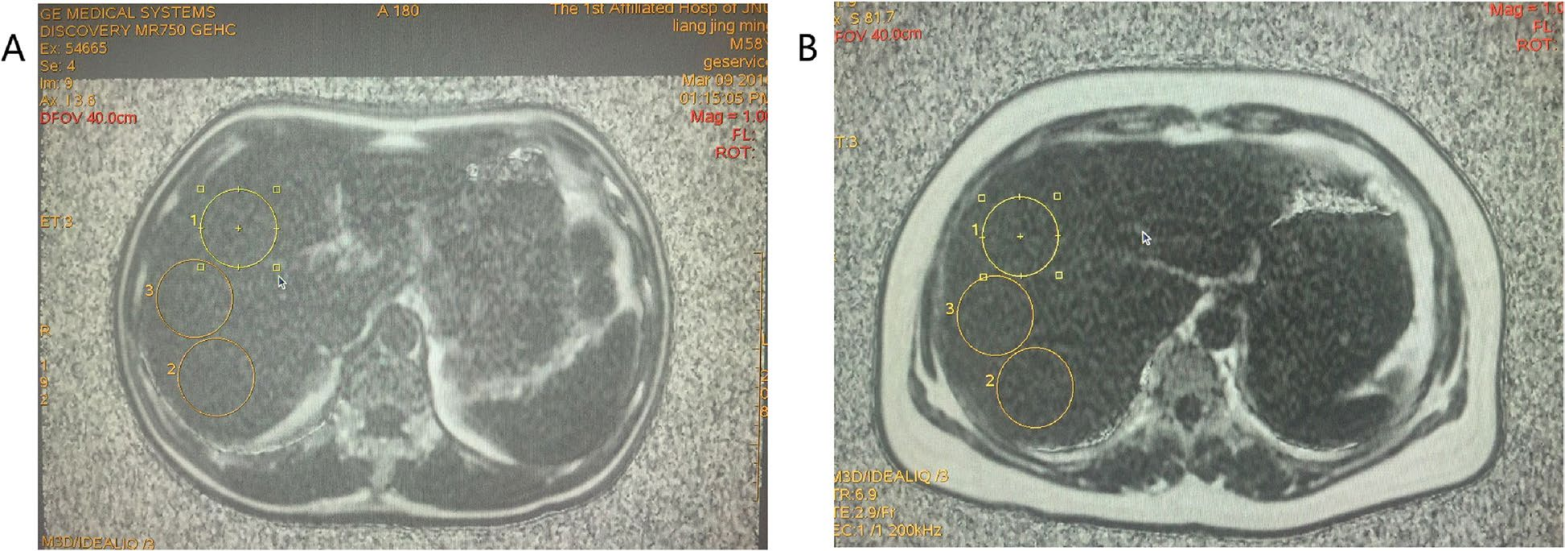
Location of the region of interest (ROI) in MRI IDEAL-IQ Sequence. **A** In-phase image of the region of interest. **B** Out-of-phase image of the region of interest

All patients underwent MRI IDEAL-IQ Sequence to measure LFC and were subjected to upper abdominal scanning by a 3.0 T MRI scanner (GE Discovery 750 Plus, GE Healthcare, USA). After preoperative fasting and water deprivation for 4–6 h, patients were placed in a supine position with phased array coils in the center of the liver. The scanning range included the top of the diaphragm to the lower margin of the liver. Parameters of IDEAL-IQ Sequence were TR 15.6 ms, TE 4.6 ms, slice thickness of 10 mm, number of echoes 6, pixel bandwidth of 111.11 kHz, field of vision 44 cm × 44 cm, matrix size 224 × 160, and flip angle of 8°. The scan was acquired during a single breath hold, lasting less than 30 s. Images were collected to obtain in-phase, anti-phase, and liver fat fractions. The region of interest (ROI) of each patient was located at the right lobe of the liver and selected in parts with more substantial composition, less intrahepatic bile duct, and blood vessels, as shown in Fig. 1. Three circular ROIs with an area of about 1200 mm were determined by two radiologists at our hospital in a cross-double-blind way, and the mean LFC of ROI was obtained.

### Chronic complications

Chronic complications associated with T2DM were mainly the following: atherosclerotic cardiovascular disease (ASCVD), diabetic nephropathy (DN), diabetic retinopathy (DR), and diabetic peripheral neuropathy (DPN). ASCVD was defined as nonfatal myocardial infarction or coronary heart disease death or fatal or nonfatal stroke [18]. The ASCVD was diagnosed by both anamnesis and medical examination. DN was diagnosed in accordance with urinary ACR ≥ 30 μg/mg and/or reduced renal function as presented by the reduced eGFR or elevated SCr [19]. DR was characterized by hard exudates, vitreous hemorrhage, cotton-wool spots, and intra-retinal microvascular changes over retinal tissue, according to the severity scale provided by the Early Treatment for Diabetic Retinopathy Study (ETDRS) and was defined as ETDRS scores ≥ 20 [20, 21]. The diagnosis of DPN was dependent on both subjective symptoms and signs of neuropathy, which was defined as at least moderate signs when the Neuropathy Disability score (NDS) was more than 6 with or without symptoms or mild signs (NDS ≥ 3) with moderate symptoms of Neuropathy symptom score ≥ 5 [22].

### Sample size calculation

We assumed that a 20% absolute difference in LFC between the two groups would be the minimally appreciable and clinically relevant difference. Thus, the current study required a sample size of at least 40 participants per group to detect the difference with a power of at least 80% at 5% significance level, estimating a dropout rate of 20%.

### Statistical analysis

Statistical analysis was carried out using the software SPSS 20.0. Data are presented as mean ± standard deviation (SD) or medians with interquartile ranges (IQR) for continuous variables and percentages (%) for categorical variables. The non-normal distribution data were logarithmically transformed into a normal distribution. To compare continuous variables, a student t-test was applied whereas categorical variables were compared using the χ2 test and a one-way analysis of variance (ANOVA) was performed to compare the difference between different groups. Multivariable logistic regression analysis was performed by estimating odd ratios (OR) and 95% confidence intervals (95% CIs) for independent risk factor analysis. The significant difference was considered as *P* < 0.05.

## Results

### Comparison of patient clinical data in NAFLD and non-NAFLD groups

There were 41 patients in the NAFLD group and 40 patients in the non-NAFLD group. The BMI, SBP, DBP, TG, ALT, AST, GGT, UA, HbA1c, FCP, 2 h CP, HOMA-IR, HOMA-IS and inflammation markers in the NAFLD group were significantly higher than those in the non-NAFLD group (*P* < 0.05). As for the chronic complications, although the incidences of ASCVD, DN, DPN, DR, and DF in the NAFLD group were higher than those in the non-NAFLD group, the difference was not statistically significant (*P* > 0.05), as shown in Table 1.

**Table 1:** Comparison of clinical parameters of patients in NAFLD and non-NAFLD groups

Variables	NAFLD group (*n* = 41)	Non-NAFLD group (*n* = 40)	*P*
Sex (male/female, n)	23/18	19/21	0.439
Age (year)	53.9 ± 10.6	56.9 ± 9.8	0.183
Diabetes duration (year)	5.4 ± 4.4	5.8 ± 4.6	0.712
BMI (kg/m^2^)	26.2 ± 4.0	23.9 ± 3.9	0.010
SBP (mmHg)	139 ± 16.7	128 ± 15.0	˂0.01
DBP (mmHg)	83 ± 12.4	75 ± 9.7	0.048
TC (mmol/L)	5.8 ± 1.3	5.5 ± 1.1	0.315
TG (mmol/L)	2.3 ± 1.9	1.6 ± 1.0	0.045
LDL-C (mmol/L)	3.1 ± 0.9	3.2 ± 1.0	0.607
HDL-C (mmol/L)	1.1 ± 0.3	1.2 ± 0.4	0.139
ApoA (mmol/L)	1.4 ± 0.3	1.4 ± 0.3	0.867
ApoB (mmol/L)	1.2 ± 0.3	1.2 ± 0.7	0.864
Lipoprotein (a) (mg/dL)	288.2 ± 161.4	282.5 ± 178.5	0.528
ALT (U/L)	49.9 ± 60.9	21.7 ± 14.7	˂0.01
AST (U/L)	31.5 ± 26.1	19.2 ± 7.4	˂0.01
GGT (U/L)	39.9 ± 30.2	28.9 ± 24.8	˂0.01
ALP (U/L)	91.7 ± 31.6	83.54 ± 25.7	0.192
UA (mmol/L)	358.9 ± 90.9	314.2 ± 88.5	0.030
TNF-α (pg/ml)	29.4 ± 1.72	21.7 ± 3.03	0.036
IL-6 (pg/ml)	41.1 ± 0.47	28.4 ± 5.27	˂0.01
IL-10 (pg/ml)	51.6 ± 1.52	66.7 ± 3.38	˂0.01
SCr (umol/L)	64.4 ± 21.8	64.0 ± 24.1	0.742
BUN (mmol/L)	5.0 ± 1.7	5.1 ± 1.3	0.787
CYC (mg/L)	1.2 ± 0.4	1.1 ± 0.3	0.741
FPG (mmol/L)	9.2 ± 2.6	9.2 ± 2.3	0.913
2 h PG (mmol/L)	18.1 ± 4.7	17.0 ± 4.2	0.269
HbA1c (%)	9.6 ± 2.2	8.6 ± 2.1	0.047
FCP (mmol/L)	1.7 ± 0.8	1.1 ± 0.7	˂0.01
2 h CP (mmol/L)	4.7 ± 2.4	3.1 ± 2.0	˂0.01
HOMA-IR	3.4 ± 1.9	2.1 ± 1.0	˂0.01
HOMA-IS	31.6 ± 20.5	21.2 ± 14.9	0.014
ASCVD (n, %)	34 (82.9)	32 (80.0)	0.735
DN (n, %)	12 (29.2)	9 (22.5)	0.487
DPN (n, %)	19 (46.3)	16 (40.0)	0.565
DR (n, %)	29 (70.7)	23 (57.5)	0.214

### Comparison of patients with different LFC

After measuring the LFC, the 81 enrolled patients were divided into four groups from high to low LFC, namely group A, group B, group C, and group D with P25, P50, and P75 as the critical value. There were 21 patients in group A with LFC (%) of 15.7 ± 7.5, 20 patients in the group B with LFC (%) of 7.2 ± 1.1, 20 patients in the group C with LFC (%) of 4.4 ± 0.5, and 20 patients in the group D with LFC (%) of 2.8 ± 0.5. After that, we compared different characteristics between these four groups. The results showed that there was no significant difference between the four groups with respect to sex, age, diabetes duration, LDL-C, HDL-C, ApoA, ApoB, lipoprotein (a), ALP, UA, SCr, BUN, CYC, FPG, 2 h PG, or HbA1c parameters (*P *> 0.05). However, there was an obvious difference in the BMI, SBP, DBP, TC, TG, ALT, AST, GGT, FCP, 2 h CP, HOMA-IR, and HOMA-IS variables between the four groups, and these indexes in groups B, C, and D were prominently lower than those in the group A (*P* <0.05). Moreover, we also noticed a significant difference in these parameters in groups C, and D when compared with group B (*P* <0.05) while we did not see any obvious difference between groups C and D (*P *> 0.05), as shown in Table 2.

**Table 2:** Comparison of patients with different LFC

Parameters	Group A (*n* = 21)	Group B (*n* = 20)	Group C (*n* = 20)	Group D (*n* = 20)	*P*
Sex (male/female, n)	15/6	8/12	12/8	7/13	0.067
Age (year)	52.0 ± 13.2	56.1 ± 6.7	56.0 ± 7.9	58.0 ± 10.9	0.259
Diabetes duration (year) (year)	5.7 ± 4.6	6.6 ± 5.3	6.4 ± 4.5	5.6 ± 4.6	0.561
BMI (kg/m^2^)	27.1 ± 4.2	25.1 ± 3.4^a^	23.0 ± 4.22^ab^	22.89 ± 3.49^ab^	˂0.01
SBP (mmHg)	146 ± 17.9	138 ± 16.1^a^	128 ± 14.4^ab^ 3^a^	127 ± 13.3^ab^	˂0.01
DBP (mmHg)	89 ± 13.5	82 ± 10.72^a^	76. ± 8.2^a^	77 ± 10.14^ab^	0.025
TC (mmol/L)	6.5 ± 1.2	5.3 ± 1.53^a^	5.5 ± 1.1^ab^	5.0 ± 1.0^ab^	0.049
TG (mmol/L)	3.5 ± 2.2	2.5 ± 0.8 ^a^	1.7 ± 1.2^ab^	1.5 ± 0.7^ab^	˂0.01
LDL-C (mmol/L)	3.3 ± 0.9	3.1 ± 1.0	3.4 ± 0.9	2.9 ± 0.9	0.359
HDL-C (mmol/L)	1.1 ± 0.3	1.2 ± 0.3	1.3 ± 0.4	1.2 ± 0.3	0.227
ApoA (mmol/L)	1.4 ± 0.25	1.49 ± 0.24	1.46 ± 0.28	0.34 ± 0.27	0.227
ApoB (mmol/L)	1.3 ± 0.2	1.1 ± 0.3	1.2 ± 0.3	1.1 ± 0.3	0.066
Lipoprotein (a) (mg/dL)	296.7 ± 166.1	282.5 ± 155.6	263.10 ± 195.1	300.40 ± 160.2	0.896
ALT (U/L)	53.7 ± 59.4	40.0 ± 62.7^a^	22.1 ± 10.0^ab^	21.4 ± 18.3^ab^	˂0.01
AST (U/L)	35.1 ± 20.1	25.5 ± 32.3^a^	18.2 ± 4.7^ab^	20.4 ± 9.3^ab^	˂0.01
GGT (U/L)	43.9 ± 35.1	34.6 ± 24.1^a^	28.0 ± 20.3^ab^	27.4 ± 28.9^ab^	˂0.01
ALP (U/L)	88.0 ± 22.9	86.1 ± 36.8	87.0 ± 27.1	80.5 ± 24.0	0.444
UA (mol/L)	359.8 ± 88.4	346.2 ± 99.9	315.8 ± 85.9	329.2 ± 99.8	0.465
SCr (umol/L)	66.1 ± 21.2	62.7 ± 22.8	60.2 ± 17.1	67.8 ± 29.5	0.722
BUN (mmol/L)	5.1 ± 1.6	4.8 ± 1.8	5.1 ± 1.3	5.1 ± 1.4	0.927
CYC (mg/L)	1.1 ± 0.3	1.2 ± 0.4	1.0 ± 0.3	1.1 ± 0.4	0.453
FPG (mmol/L)	9.4 ± 3.2	9.1 ± 1.8	9.5 ± 2.5	8.8 ± 2.1	0.183
2 h PG (mmol/L)	19.0 ± 5.4	17.0 ± 3.6	17.9 ± 4.5	16.1 ± 3.8	0.183
HbA1c (%)	9.7 ± 2.5	9.6 ± 1.9	9.8 ± 2.6	8.6 ± 2.2	0.749
FCP (mmol/L)	1.9 ± 0.8	1.5 ± 0.9^a^	1.1 ± 0.6^ab^	1.1 ± 0.7^ab^	0.022
2 h CP (mmol/L)	5.5 ± 2.6	4.2 ± 2.2^a^	2.9 ± 1.9^ab^	3.2 ± 2.2^ab^	˂0.01
HOMA-IR	3.9 ± 2.1	3.0 ± 1.7^a^	2.2 ± 0.9^ab^	2.0 ± 1.2^ab^	˂0.01
HOMA-IS	35.9 ± 22.4	27.1 ± 17.8^a^	21.1 ± 17.9^ab^	20.3 ± 11.6^ab^	0.030

^a^*P* < 0.05 represents comparison of groups B, C, and D with group A; ^b^*P* < 0.05 represents comparison of groups C and D with group B

### Correlation of T2DM with chronic complications

#### Influencing factors of T2DM complicated with ASCVD

The presence of ASCVD complication (none for 0, yes for 1) was set as the dependent variable, and *P* = 0.05 and *P* = 0.10 were adopted as the test criterion of the introduced variable and excluded variable, respectively. After univariate unconditional logistic regression analysis, diabetes duration, BMI, SBP, TC, TG, HbA1c, HOMA-IR, and LFC were found to be influential factors associated with ASCVD complications. Based on these influential factors, *P* = 0.10 and *P* = 0.15 were adopted as the test criterion of the introduced variable and excluded variable to conduct a binary logistic regression analysis, respectively. As shown in Table 3, diabetes duration, TC, HOMA-IR, and LFC were among the risk factors for ASCVD complications in T2DM patients (*P* < 0.05).

**Table 3:** Logistic regression analysis of influencing factors of T2DM complicated with ASCVD

Variables	B-value	S.E	*P*-value	OR (95% CI)
Diabetes duration	2.186	0.841	˂0.01	8.902 (1.711, 46.322)
TC	1.268	0.637	0.046	3.554 (1.020, 12.374)
HOMA-IR	1.609	0.818	0.049	5.000 (1.006, 24.851)
LFC	0.648	0.261	0.013	1.911 (1.146, 3.181)

#### Influencing factors of T2DM complicated with DN

Similar to ASCVD, the presence of DN complication was set as the dependent variable, and univariate logistic regression analysis showed that diabetes course, SBP, TG, LDL-C, and HOMA-IR were the influencing factors of DN complication with the introduced variable test criterion of *P* = 0.05 and excluded variable test criterion of *P* = 0.10. After binary logistic regression analysis with the introduced variable test criterion of *P* = 0.10 and excluded variable test criterion of *P* = 0.15, diabetes duration, TG, and LDL-C were found to be the risk factors for DN complication in T2DM patients (*P* < 0.05), as shown in Table 4.

**Table 4:** Logistic regression analysis of influencing factors of T2DM complicated with DN

Variable	B-value	S.E	*P*-value	OR (95% CI)
Diabetes duration	0.151	0.060	0.012	1.163 (1.034, 1.308)
TG	0.459	0.193	0.017	1.582 (1.084, 2.308)
LDL-C	0.609	0.302	0.044	1.838 (1.016, 3.325)

#### Influencing factors of T2DM complicated with DR

The presence of DR complication was set as the dependent variable, the univariate logistic regression analysis showed that diabetes duration and SBP were the influencing factors of DR complication, and binary logistic regression analysis indicated that diabetes duration and SBP were the risk factors for DR complication in T2DM patients (*P* < 0.05), as shown in Table 5.

**Table 5:** Logistic regression analysis of influencing factors of T2DM complicated with DR

Variable	B-value	S.E	*P*-value	OR (95% CI)
Diabetes duration	0.498	0.139	˂0.01	1.645 (1.252, 2.162)
SBP	0.042	0.020	0.032	1.043 (1.004, 1.085)

## Influencing factors of T2DM complicated with DPN

The presence of DPN complication was set as the dependent variable, and the univariate logistic regression analysis showed that diabetes duration and SBP were the influencing factors of DPN complication. Additionally, binary logistic regression analysis indicated that diabetes duration and SBP were the risk factors for DPN complication in T2DM patients (*P* < 0.05), as shown in Table 6.

**Table 6:** Logistic regression analysis of influencing factors of T2DM complicated with DPN

Variable	B-value	S.E	*P*-value	OR (95% CI)
Diabetes duration	0.100	0.052	0.055	1.105 (0.998, 1.224)
SBP	0.029	0.015	0.060	1.029 (0.999, 1.060)

## Discussion

Compared with qualitative analysis, quantitative analysis of liver fat content can better reflect the fat deposition degree to explore the correlation with metabolic disorders, such as diabetes, and its chronic complications. In recent years, many studies have revealed the correlation of LFC with various metabolic disorders but most of these studies were based on the qualitative diagnosis of visceral fat (mainly liver fat), namely the existence of NAFLD [23, 24]. Although very few studies were conducted on the quantitative diagnosis of LFC [25, 26], but in all these studies computed tomography (CT) quantification was the main technology, which could not precisely quantify LFC and MR was less used. Moreover, most correlation studies are performed on single chronic complications, such as ASCVD and/or diabetic nephropathy, while correlation studies on overall chronic complications of T2DM are relatively rare. In this paper, we take the opportunity to precisely and quantitatively measure LFC using the MRI IDEAL-IQ Sequence to explore the relationship of liver fat content with systemic metabolism and the correlation of various chronic complications with T2DM.

NAFLD is closely associated with T2DM, and some studies believe that insulin resistance may serve as a bridge between the etiology and clinical features of NAFLD and may attribute to the main risk factors for the development of NAFLD [27]. Visceral obesity, T2DM, dyslipidemia, and arterial hypertension are the phenotypic expression of NAFLD, and the presence of one or more of these conditions increases the risk of developing NAFLD [28]. In our study, the BMI, SBP, DBP, TG, ALT, AST, GGT, UA, HbA1c, FCP, 2 h CP, HOMA-IR, and HOMA-IS in T2DM patients with NAFLD were significantly higher than those without NAFLD (*P* < 0.05), suggesting that T2DM with NAFLD patients are more prone to metabolic abnormalities and insulin resistance, which is consistent with other studies [24, 29]. However, there was no significant difference in TC, TG, LDL-C, and H-LDL between the NAFLD and non-NAFLD groups, which may be largely related to the utilization of lipid-lowering drugs in clinical practice impacting the results.

MRI IDEAL-IQ Sequence is an ideal examination method to evaluate the degree of liver fat infiltration [30]. In the present study, we utilized this technology to measure LFC and then classified patients into different groups based on the LFC. Upon investigation, we found that the patients with different LFC showed a significant difference in BMI, SBP, DBP, TC, TG, ALT, AST, FCP, 2 h CP, HOMA-IR, and HOMA-IS variables. Our results also indicated that the higher the difference in LFC among groups, the greater the difference in metabolic indexes, and the more serious the metabolic disorder, which further supports the results of the NAFLD group to some extent.

T2DM is generally characterized by insulin resistance and is associated with multiple chronic complications including retinopathy, nephropathy, neuropathy, diabetic foot, and other vascular complications [31]. It has been reported that NAFLD is an independent risk factor for diabetes-associated complications and the likelihood of developing these complications increases in T2DM patients complicated with NAFLD [32]. In our study, the incidences of ASCVD, DN, DR, and DPN in the NAFLD group were higher than those in the non-NAFLD group, but the difference was not statistically significant, which is in agreement with the results of previous studies [33, 34]. Based on this, the correlation between LFC and chronic complications was further explored by logistic regression analysis. The results showed that diabetes duration was a common risk factor for ASCVD, DN, DR, and DPN. In addition, TC, HOMA-IR, and LFC were the independent risk factors for T2DM complicated with ASCVD. Moreover, TG and LDL-C were the main risk factors for DN while SBP was closely related to the occurrence and development of DR and DPN, which can be considered the main risk factor.

### Strengths and limitations

The current study offers novel information on the potential use of MRI IDEAL-IQ Sequence technology to determine liver fat content in T2DM patients. It is inspiring to propose that, unlike other noninvasive techniques like ultrasonography or computed tomography, the MRI IDEAL-IQ Sequence method measures the percentage of liver fat and provides highly accurate quantitative measurements of the amount of liver fat, allowing grading of disease severity and its correlation with other chronic complications.

Although the results of the present study provide some interesting information, this study was limited by some issues. The sample size was not sufficient as the detection rate of chronic complications had no significant difference between the NAFLD group and the non-NAFLD group, even when patients were classified according to LFC by quartile method. Accordingly, lipid-lowering intervention is often adopted in clinics for treating T2DM patients, and the lipid-lowering drugs are not stopped before the examination, which also might have affected the outcomes of this study. Thus, it is necessary and important to increase the sample size and limit the lipid-lowering intervention, in order to ensure the accuracy of reported data. Likewise, we did not examine the correlation between hepatic fat content and inflammatory markers and diabetic complications that need further investigation. Subsequently, the medication status of the participants was not included in the reported data thus limiting the implication of this study. Moreover, due to variable LFC deposition in different parts of the liver, further investigation is needed to highlight the correlation of LFC in different parts of the liver with systemic metabolism and chronic complications in T2DM patients.

## Conclusions

In summary, the results of the present study demonstrate that LFC can be precisely determined by MRI IDEAL-IQ Sequence, which has a positive correlation with systemic metabolic characteristics, and diabetic complications. Thus, in clinical practice, in addition to measuring blood glucose levels, liver fat content should also be evaluated by MRI IDEAL-IQ Sequence technology for early diagnosis and effective intervention of T2DM and fatty liver-associated diseases.

## Data Availability

The datasets generated during and/or analyzed during the current study are available from the corresponding author upon reasonable request.
